# Determinants of Iran’s *Bilateral* *Intra-industry Trade* in Pharmaceutical Industry

**Published:** 2018

**Authors:** Siamak Aghlmand, Bahlol Rahimi, Hamidreza Farrokh-Eslamlou, Bahram Nabilou, Hassan Yusefzadeh

**Affiliations:** a *Determinants of Health Research Center, Urmia University of Medical Sciences, Urmia, Iran. *; b *Department of Health Information Technology, School of Para Medicine, Urmia Medical Sciences University, Urmia, Iran.*; c *Reproductive Health Research Center, School of Public Health, Urmia University of Medical Sciences, Urmia, Iran. *; d *Determinants of Health Research Center, Urmia University of Medical Sciences, Urmia, Iran. *; e *Department of Public Heath, School of Health, Social Determinants of Health Research Center, Urmia Medical Sciences University, Urmia, Iran.*

**Keywords:** Intra-industry trade, Pharmaceutical products, Bilateral, Gravity model, Iran

## Abstract

Among non-oil and in trade arena, drug has always been strategic importance and most government especially industrialized countries pay special attention to its production and trade issues. Thus, having a comprehensive view from economic perspective to this section is essential for suggesting intervention. This was a descriptive-analytical and panel study. In this study, gravity model is used to estimate Iran’s bilateral intra-industry trade in pharmaceutical products in the 2001-2012 periods. To illustrate the extent of pharmaceutical’s intra-industry trade between Iran and its major trading partners, the explanatory variables of market size, income, factor endowments, distance, cultural contributions, and similarities and also special trade arrangements have been applied. Analysis of factors affecting Iran’s bilateral intra-industry trade in pharmaceutical industry showed that the average GDP and cultural similarities had a significant positive impact on Iran’s bilateral IIT, while the difference in GDP has a negative and significant effect. Coefficients obtained for the geographical distance and the average ratio of total capital to the labor force is not consistent with theoretical expectations. Special trade arrangements did not have significant impact on the extent of bilateral intra-industry trade between Iran and its trading partners. The knowledge of the intra-industry trade between Iran and its trade partners make integration between the countries. Factors affecting this type of trade pattern underlie its development in trade relationship. Therefore, the findings of this study would be useful in helping to develop and implement policies for the expansion of the pharmaceutical trade.

## Introduction

Emphasis on non-oil and high-technology exports is realized through export of goods such as pharmaceutical products which are considered a basic human need. Thus, governments pay special attention to manufacturing and trade of pharmaceutical products along with other goods such as food and clothing and include in import, export, and support baskets as important commodities (1).

Pharmaceutical products are considered as commodity products not only in Iran but also throughout the world. Given the unique features and healthcare function of pharmaceuticals, no alternative has been found to replace them (2).

Countries that already have a good position in the pharmaceutical industry seek to facilitate trade through removing unnecessary trade barriers because today, self-sufficiency in production is not considered as an indicator of one nation’s supremacy. A manufacturer who does not pay attention to foreign markets and has restricted its perspective to the domestic market will undoubtedly have a short life and, before long, will lose its market share to its international competitors even in the domestic market (3).

Iran is increasingly becoming integrated with the global economy. Thus, achieving a successful economic performance in the pharmaceutical industry is of utmost importance for its survival (4).

According to the statistics provided by World Health Organization (WHO) and World Trade Organization (WTO), the pharmaceutical industry ranks second among the world’s 52 most profitable industries. This statistics suggests that the global pharmaceutical market is worth $1200 billion. Iran’s pharmaceutical market has an economic value of around $4 billion (5). Therefore, pharmaceuticals can be considered as strategic and profitable commodities (6). In Iran, enough attention was not paid to the pharmaceutical industry and its high value-added. As a result, when compared with competing countries, the Iranian pharmaceutical industry is not sufficiently developed in terms of scale and dimensions (7). Maintaining human health, reducing consumption of pharmaceuticals, promoting health, the promotion of health at a global level, reducing foreign currency expenditure, effective employment generation, etc. are some of the factors necessitating the development of pharmaceutical industry in Iran (8). Analyzing the determinants of Intra-Industry Trade (IIT) is a relatively new approach in field of international economic research. IIT is an empirical phenomenon that has been found in international trade over recent years. IIT occurs when a country simultaneously exports and imports goods or services produced by the same industry (9). It is different from inter-industry trade, in which a country specialises in the production of a good or service and exports it, in exchange for a different good or service for which it has no comparative advantage (10).

**Table 1:** Bilateral IIT in Pharmaceuticals: Iran and its 23 Trade Partners (dependent variable =${\mathrm{LIIT}}_{\mathrm{ij}}$**).**

**Variable**	**Expected sign**	**coefficients**	**t-Statistic**	**Std. Error**	**Prob.**
C		-113.9028	-8.620020	13.213	0.0000
Average GNP (AGNP_ij_)	+	6.591030	11.18803	0.589	0.0000
Difference in GNP (DGNP_ij_)	-	-1.629270	-9.227040	0.176	0.0000
Average per capita GNP (APGNP_ij_)	+	15.75204	4.319792	3.646	0.0000
Difference in per capita GNP (DPGNP_ij_)	-	-1.566467	-1.824279	0.8586	0.0713
Average capital-labour ratio (AKL_ij_)	+	-9.500001	-10.02071	0.948	0.0000
Difference in capital-labour ratio (DKL_ij_)	-	-0.709400	-1.903903	0.372	0.0600
Distance (DIS_ij_)	-	0.592565	2.305784	0.256	0.0233
Cultural similarities (CULT_ij_)	+	0.382708	2.979944	0.1284	0.0037
Special trading arrangement (TRRA_ij_)	+	0.149014	1.239733	0.1201	0.2182
R-squared	0.9313				
Adjusted R-squared	0.9248				
F-statistic	141.75				
Durbin-Watson stat	1.96				
Schwarz criterion	2.5698				
Akaike info criterion	2.4722				

The IIT phenomenon has significant political implications. For example, IIT has reduced the need for support since it involves both sides of a foreign trade, namely, imports and exports. Furthermore, compared to inter-industry trade, IIT entails lower adjustment costs following trade liberalization. Trade liberalization leads to the transfer of production factors between sectors, which, in turn, inflict costs on the economy. The nature of IIT, which involves commodity groups with the same factor proportion, leads to a reduction of adjustment costs. Specifically, countries with a sufficiently similar balance benefit from trade liberalization and face less adjustment issues compared to the standard circumstances (conventional theories) (11). Since foreign-exchange resources are considered as one of the key determinants of development and the pharmaceutical industry plays a significant role in encouraging the flow of foreign currency, investigating the factors influencing Iran’s bilateral IIT in pharmaceuticals seems necessary. In this regard, the pharmaceutical industry will develop through pharmaceutical export promotion. Not only does increased export growth have a direct impact on pharmaceuticals but also it will enhance labor and capital inputs’ productivity and promote economic prosperity through export promotion (12).

This research is the first study analyzing the factors influencing Iran’s bilateral IIT in the pharmaceuticals. It attempted to find which factors play a more important role in determining and explaining bilateral IIT in Iran’s pharmaceutical industry.

*Methods*

The most widely used IIT scale is an index introduced by Grubel and Lloyd. According to this scale, inter-industry trade is the measured based on the proportion of absolute value of imports and exports |X_i_-M_i_| to the total value of trade (X_i_+M_i_) in the industry. On the other hand based on this index, IIT is measured based on proportion of the remaining value of the total trade after being subtracted from the net trade to the total value of trade in the industry. Grubel-Lloyd index, in its abbreviated form, calculates IIT by subtracting it from 1 (13).

*GL*_i _is the Grubel-Lloyd index and *X*_i_ and *M*_i_ are *i* industry’s export and import values, respectively.

If the entire trade is IIT, *GL*_i_ index is equal to 100. In other world, under such circumstance, imports and exports of the same industry are equal. On the other hand, the index will be zero if there is no IIT in the specified industry. In other words, the entire trade in the specified industry is inter-industry trade.

The gravity model is a method used for studying potential economic integration among countries, evaluating potential trade capacity, measuring effects of deviation and trade creation, calculating the variables influencing the volume of trade and, subsequently, assessing trade partners’ features based on the same variables (14).

It is assumed that bilateral IIT of pharmaceuticals between Iran and its trade partners is dependent on the following explanatory variables:

1. Market size: Market size is calculated based on the gross national product (GNP). *AGNP*_ij_ and *DGNP*_ij_ are the average *GNP* and the absolute difference in *GNP* between Iran and country *j*, respectively. When market sizes of two countries are equal, products tend to be differentiated. As a result, the volume of IIT is expected to be larger. In other words, the higher the average GNP, the larger the volume of IIT between the two countries. On the other hand, greater absolute difference between the market sizes is expected to negatively influence IIT (15).

2. Income: GNP per capita is used to calculate income of a country. *AGNP*_ij_ and *DGNP*_ij _ are the average per capita *GNP* and the absolute difference in per capita *GNP* between Iran and country *j*, respectively. Income influences on consumers’ tastes and priorities. An increase in income leads to an increase in demand for differentiated products. Thus, it is assumed that the larger the average per capita income of Iran and country *j*, the greater the share of IIT. In contrast, the great absolute difference in income between Iran and country *j*, the smaller the share of IIT (16).

3. Factor Endowment: Capital-labor ratio is an indicator of a country’s relative factor endowments. *AKL*_ij_ and *DKL*_ij_ are the average of and the absolute difference in capital-labor ratio between Iran and country *j*, respectively. Differentiated products are more likely to be capital intensive. Thus, manufacturing of differentiated products will increase relevant to the capital endowments. Both countries have high overall capital-labor ratios and will consequently be more likely to manufacture differentiated products and involve in high levels of IIT. The average total capital-labor ratio and the absolute difference in the ratio between Iran and country *j* are expected to have a positive and negative impact on IIT in pharmaceuticals between Iran and Country *j*, respectively (17).

4. Distance (*DIS*_ij_): Distance is defined as the geographical distance between Iran and country *j* in Kilometers. The distance between Iran and its trade partners has a significant impact on IIT. A country’s geographical features determine production costs and transit duration. Given the transit costs, countries located farther from Iran consider it as having unfavorable IIT conditions. As a result, the distance between Iran and country j is expected to have a negative impact on IIT (18).

5. Cultural similarities (*CULT*_ij_): *CULT*_ij_ shows the cultural similarities (common language and religion) between Iran and country *j*. The similarities between Iran and its trade partners are expected to have a positive impact on the volume of IIT. *CULT*_ij _ will be set to 1 for countries with common language and religion. For other countries, *CULT*_ij _ is set to 0 (19).

6. Special trading arrangements (*TRRA*_ij_): *TRRA*_ij_ shows the special trading arrangements between Iran and Country *j*. A *TRRA*_ij _ with a value of 1 indicates country j’s close economic ties with Iran (Afghanistan, Iraq, China, etc.). On the other hand, A *TRRA*_ij _ with a value of 0 is related to Iran’s economic ties with its other trade partners. Special trading arrangements are expected to have a positive impact on IIT in pharmaceuticals (20).

In order to create the appropriate functional form, log-linear and log-log forms are estimated and tested based on diagnostic statistics:

Log-linear form:

LIIT_ij_ = β_0_ + β_1_AGNP_ij_ + β_2_DGNP_ij_ + β_3_APGNP_ij_ + β_4_DPGNP_ij_ + β_5_AKL_ij_ + β_6_DKL_ij_ + β_7_DIS_ij_ + β_8_CULT_ij_ + β_9_TRRA_ij_ + u_ij_

Log-log form:

LIIT_ij_ = β_0_ + β_1_LAGNP_ij_ + β_2_LDGNP_ij_ + β_3_LAPGNP_ij_ + β_4_LDPGNP_ij_ + β_5_LAKL_ij_ + β_6_LDKL_ij_ + β_7_LDIS_ij_ + β_8_CULT_ij_ + β_9_TRRA_ij_ + u_ij_

The analysis’s dependent variable is the standard Grubel-Lloyd index which is limited to a theoretical range of 0-100. Thus, the logistic conversion of Grubel-Lloyd index will be applied as follows:

 LIIT_iw_= log $\frac{\left[{\mathrm{IIT}}_{\mathrm{iw}}\right]}{\left[100-{\mathrm{IIT}}_{\mathrm{iw}}\right]}$

*β*_0_is the y-intercept statement. *β*_1_, *β*_2_, *β*_3_, *β*_4_, *β*_5_, *β*_6_, *β*_7_, *β*_8_, and *β*_9_ are the coefficients of independent variables. *u*_ij_ is the error statement. *β*_1_, *β*_3_, *β*_5_, *β*_8_ and *β*_9 _are expected to be positive. *β*_2_, *β*_4_, *β*_6_ and *β*_7_ are expected to be negative.

In order to find an appropriate functional form for a model of bilateral IIT in pharmaceuticals in Iran, log-linear and log-log forms were tested using diagnostic statistics (normality, variance heterogeneity, functional form misspecification (RESET), and serial correlation). The aforementioned tests were performed in Eviews. In addition, quarterly statistics of 2001-2012 were used to calculate the function (21, 22). As a result, the total number of observations for each variable to be used in the calculation of the function of Iran’s bilateral IIT in pharmaceuticals was 276. The augmented Dickey-Fuller test (ADF) was performed to investigate the existence of unit root in each data series. Since the variables are non-stationary, conducting a cointegration test was necessary. Engle-Granger test confirmed long-run relationship between the variables.

## Results

The results of diagnostic tests show that log-log form is the preferable functional form for the model of Iran’s bilateral IIT in pharmaceuticals. The regression results for this model are presented in Table 1. As shown in the below table, the coefficients of average GNP, difference in GNP, average per capita GNP, difference in per capita GNP, average capita-labor ratio, difference in capital-labor ratio, geographical distance, and cultural similarities statistically had a significant effect on Iran’s bilateral IIT in pharmaceuticals. However, the coefficients of geographical distance and the average capital-labor ratio did not have the expected sign. The coefficient of special trading arrangements was not statistically significant, yet it had the expected sign.

As expected, Iran’s bilateral IIT in pharmaceuticals is positively related to the average market size (LAGNP_ij_), but negatively related to the market size difference (LDGNP_ij_) at a significance level of 0.05. These results and the similarities between Iran and its trade partners in terms of market size, economic foundation, and technological complexity confirm the large volume of bilateral IIT in pharmaceuticals. In contrast, the market size differences between Iran and its trade partners lead to a lower volume of bilateral IIT in pharmaceuticals. Since the GDP is introduced as economic base in each economic system, so an increase in GDP leads to improve in the country›s ability to produce pharmaceutical products and the volume of trade between two countries increase.

Furthermore, the average per capita GNP (LAPGNP_ij_) and the per capita GNP difference (LDPGNP_ij_) have significantly direct and inverse impact on Iran’s bilateral IIT in pharmaceuticals (at a 0.05 and 0.07 significance level). This confirms that the higher income levels in the trading partners of each country have positive effect on bilateral intra industry trade between Iran and the major trading partners because there is more demand for imports in these countries.

To the contrary of what was expected, the average capita-labor ratio (LAKL_ij_) is negatively related to Iran’s bilateral IIT in pharmaceuticals. In other words, the variable does not have a significant impact on the bilateral IIT in pharmaceuticals between Iran and its trade partners. However, the coefficient of the capital-labor ratio difference (LDKL_ij_) is both negative and significant (at a significance level of 0.06), indicating that the capital-labor ratio difference and Iran’s bilateral IIT in pharmaceuticals are inversely related.

The results of the preceding table showed that despite its significance (at a significance level of 0.05), geographical distance (LDIS_ij_) does not produce the expected sign. This is not compatible with the theoretical hypothesis of the model. This incompatibility might be due to the countries’ specific features, social structures or transit networks. Obviously, given the high transit costs, the geographical distance between Iran and its trade partners will lead to a decrease in the volume of Iran’s bilateral IIT in pharmaceuticals.

In addition, the coefficient of cultural similarities (CULT_ij_) is both significant (at a significance level of 0.06) and positive. This means that Iran and other countries with common language and religion will engage in more bilateral IIT in pharmaceuticals. Furthermore, the coefficient of special trading arrangements (TRRA_ij_) is both insignificant and positive. This result might be due to the limited observations that measure the special trading arrangements between Iran and its trade partners. Thus, close economic ties with other countries had a positive impact on Iran’s bilateral IIT in pharmaceuticals.

Each one percent increase in the average market size, the market size difference, the average per capita GNP, the per capita GNP difference, the average capital-labor ratio, the capital-labor difference, distance, cultural similarities, and special trading arrangements led to an average 6.59, - 1.6, 15.75, - 1.56, - 9.5, - 0.7, 0.59, 0.38, and 0.14 percent increase in bilateral IIT in pharmaceuticals between Iran and its trade partners. In this study, an increase in the average capital-labor ratio and geographical distance, respectively, decreased and increased the volume of Bilateral IIT. This is in contrast to theoretical expectations.

The coefficient of determination (R^2^) of the model was 0.93, which indicates the explanatory power of the regression is very good. In other words, 0.93% changes in bilateral IIT in specified model are explained by country-specific determinants of bilateral IIT.

## Discussion

Pharmaceuticals are the foundation, the key axis, and, in many cases, the final point of the entire healthcare and medical operations. Pharmaceuticals complete the cycle of health system in all countries. Pharmaceutical products are classified as one of the high-tech based industries. The neighboring countries, Southeast Asian countries (CIS), and some African countries are the major export destinations for Iran’s pharmaceuticals.

The results showed that the country’s average GNP and cultural similarities had a significantly positive impact on Iran’s bilateral IIT in pharmaceuticals, while the GNP difference had a significantly negative impact on Iran’s bilateral IIT in pharmaceuticals. In other words, it is more likely for Iran to engage in bilateral IIT in pharmaceuticals with countries with a similar market size and common language and religion. In contrast, it is unlikely for Iran to engage in bilateral IIT in pharmaceuticals with countries that have market sizes different from Iran’s. Thus, economic size is one of important explanatory variables of IIT in pharmaceuticals between Iran and its trade partners. A larger economic size points to broader possibilities for product differentiation (due to the greater variety of preferences and the possibility of wider use of benefits of economies of scale) and larger volume of bilateral IIT. Accordingly, it is recommended that the Iran’s trade flows in pharmaceuticals be directed toward countries with larger economic size.

Moreover, the coefficient obtained for the geographical distance variable was not compatible with the theoretical expectations. This is probably due to transit network’s structure. The results also indicated that the total average of capital-labor ratio did not have a positive impact on Iran’s bilateral IIT in pharmaceuticals. It was also statistically confirmed that there was no positive relationship between bilateral IIT in pharmaceuticals and special trading arrangements (in the form of regional trading arrangements between Iran and its trade partners). This could be due to the limited number of observations used in the analysis. 

The average per capita GNP (per capita income) and the per capita GNP difference had positive and negative impacts on Iran’s bilateral IIT in pharmaceuticals, respectively. In other words, while deciding on the trade partnerships, the different structures of countries should be considered in terms of supply (difference in production factor endowments) and demand (difference in demand structure and consumers’ preferences). Considering the supply dimension, difference in endowments of the production factors lead to reduction in IIT. Taking the demand dimension into consideration, countries with similar income structures have similar demand structures, trade similar commodities and have relatively larger volumes of trade In this regard, while deciding on trade partners, the level of development and the income distribution should be taken into consideration. 

Considering the model of bilateral IIT in pharmaceuticals, we can refer to the results of the single research study conducted in this field. Chuankamnerdkarn has investigated the models and factors affecting Australia’s international trade in pharmaceuticals. The results showed that Australia seems to be more engaged in IIT with countries with similar market size and common language. National income, the relative factor endowments (capital-labor ratio), and special trading arrangements did not significantly influence the bilateral IIT in pharmaceuticals between Australia and its trade partners. However, Australia seems to face limitations in IIT due to the large geographical distances between this country and its trade partners (23). In Iran, no study has been carried out to investigate bilateral IIT in pharmaceuticals. However, similar national and international studies have examined bilateral IIT in other industries. In most cases, a positive relation was found between bilateral IIT and trade liberalization, economic size, per capita income, human capital, and foreign direct investment (FDI). On the other hand, a reverse relationship was found between bilateral IIT and the two variables including geographical distance and trade barriers. The results of the present study are largely similar to these results (3, 4, 16, 20, 24-29).

The research results can play a valuable role in helping pharmaceutical firms and relevant policy-makers in their pharmaceutical trade decisions. In order to be more prepared for integration into the global economy, special attention should be paid to bilateral IIT in pharmaceuticals. In other words, optimizing Iran’s trade flows in pharmaceuticals requires an objective function in which is affected by the aforementioned components. This will, in turn, lead to a reduction in trade costs.
